# U.S. States’ COVID-19 physical distancing policies and working-age adult mental health outcomes

**DOI:** 10.1016/j.pmedr.2023.102370

**Published:** 2023-08-18

**Authors:** Shannon M. Monnat, David C. Wheeler, Emily Wiemers, Yue Sun, Xinxin Sun, Douglas A. Wolf, Jennifer Karas Montez

**Affiliations:** a Department of Sociology, Syracuse University, Syracuse, NY, USA; b Center for Policy Research, Syracuse University, Syracuse, NY, USA; c Lerner Center for Public Health Promotion and Population Health, Syracuse University, Syracuse, NY, USA; d Department of Biostatistics, Virginia Commonwealth University, Richmond, VA, USA; e Department of Public Administration and International Affairs, Syracuse University, Syracuse, NY, USA; f Aging Studies Institute, Syracuse University, Syracuse, NY, USA; g Center for Aging and Policy Studies, Syracuse University, Syracuse, NY, USA

**Keywords:** COVID-19, State policies, Mental health, Stay-at-home orders, Working-age adults

## Abstract

In the early months of the COVID-19 pandemic, states enacted multiple policies to reduce in-person interactions. Scholars have speculated that these policies may have contributed to adverse mental health outcomes. This study examines potential associations between states’ COVID-19 physical distancing policies and working-age (18–64) adults’ self-reported mental health. Mental health outcomes (depression, anxiety, worsened mental health, and sought treatment for anxiety or depression) are from the National Wellbeing Survey collected from working-age adults in the United States (U.S.) February 1 to March 18, 2021 (N = 3,804). Data on 12 state policies are from the COVID-19 U.S. State Policy Database. Analyses included logistic regression and Bayesian group index modeling, which identified sets, or “bundles,” of policies that were associated with each mental health outcome. Multiple policies (both separately and in bundles) were associated with adverse mental health outcomes, with certain policies (closures and curfews on retail and other businesses) being particularly important. A one-month increase in exposure to respective model-derived physical distancing policy bundles was associated with a 36% increase in the odds of reporting that COVID-19 worsened one’s mental health (odds ratio [OR] = 1·36; 95% credible interval [CRI] = 1·01 to 1·80), a 6% increase in the odds of meeting the clinical threshold for anxiety (OR = 1·06; CRI = 0·99 to 1·16), and a 15% increase in the odds of seeking treatment for anxiety or depression (OR = 1·15; CRI = 1·02 to 1·49). To accurately understand the role of states’ COVID-19 policies on mental health during the pandemic, researchers must consider how collections of policies might influence outcomes.

## Highlights

• States enacted several physical distancing policies during the COVID-19 pandemic.
• Multiple policies were associated with adverse mental health outcomes.
• Business curfews and retail and restaurant closures had the strongest associations.

## 1 Introduction

In the early months of the COVID-19 pandemic, several U.S. states enacted physical distancing policies, such as stay-at-home orders and business closures, aimed at reducing coronavirus spread by reducing in-person interactions. Scholars have speculated that these policies may have contributed to adverse mental health outcomes, in what has been called a ‘parallel pandemic’ (Yao et al., 2020).

U.S. states varied in their enactment of specific policies, combinations of policies, timing of initiation, and duration. For example, although 44 states implemented a stay-at-home order, durations varied from 25 to 313 days (Raifman et al., 2020). This variation provides an opportunity to examine potential associations between policy exposure and mental health outcomes. Physical distancing policies could contribute to adverse mental health outcomes in many ways, including disruptions to employment and income, health care access, social relationships, and family responsibilities, as well as feelings of loss of freedom, helplessness, loneliness, and anger. Working-age adults may have been particularly affected due to their joint responsibilities of work and family caretaking (Czeisler et al., 2020).

Research conducted during the early months of the pandemic found evidence of adverse impacts to mental health, such as increasing self-reported anxiety, suicidal ideation, and substance use (Czeisler et al., 2020, Brodeur et al., 2021, Coley et al., 2022, Ettman et al., 2020, Scripts, 2020, French et al., 2020, Killgore et al., 2020, McPhee et al., 2020, Twenge and Joiner, 2020, Twenge and Joiner, 2020). In a review of the psychological effects of quarantine early in the pandemic, most studies reported adverse impacts, including post-traumatic stress disorder symptoms, confusion, and anger (Brooks et al., 2020). A study comparing U.S. Google searches on wellbeing-related terms before and after stay-at-home orders in the U.S. found large increases in search intensity for boredom, loneliness, worry, and sadness (Brodeur et al., 2021). Studies based on samples collected in the early months of the pandemic (e.g., March and April 2020) found that being under a stay-at-home order was associated with greater health anxiety, financial worry, and loneliness (Tull et al., 2020), more symptoms of depression, anxiety, acute stress, and insomnia (Marroquín et al., 2020), and lower self-reported functional wellbeing (Barrett et al., 2021). However, a study using data collected from April 2020 to March 2021, found no significant association for stay-at-home orders or restaurant closures on anxiety and depression (Coley et al., 2022).

Research to date has tended to report on the effects of single policies (most commonly stay-at-home orders). Yet, states did not enact just one policy. States enacted multiple policies that restricted different types of activities, such as stay-at-home orders *and* non-essential business closures *and* day care closures. We refer to such sets of correlated policies that restricted different types of activities within the same state as “policy bundles.” The impact of exposure to multiple physical distancing policies (policy bundles) may be larger than the impact of a single policy.

The purpose of this study was to examine potential associations between state policies that restricted in-person interactions and U.S. working-age adults’ self-reported mental health outcomes. We advance the literature on this topic by a) including a more comprehensive array of policies than what has been done in previous studies; b) estimating how individual policies are associated with mental health; c) estimating how sets, or “bundles”, of policies are associated with mental health; and d) assessing the importance of each individual policy to the policy bundles.

## 2 Materials and methods

### 2.1 Study design and population

In this cross-sectional study, outcomes came from the 2021 National Wellbeing Survey (NWS), a demographically representative geocoded survey of 4,014 U.S. working-age (18–64) adults collected in February and March 2021, approximately one year after the pandemic took hold in the U.S. (Monnat and Rhubart, 2021). The NWS includes respondents from all states and the District of Columbia. The survey was administered using Qualtrics Panels, an online survey platform that maintains a database of several million U.S. adults who volunteer to participate in surveys. Online panels are increasingly used in social science research due to efficiency, cost, timeliness, and data quality (Hays et al., 2015). Recruitment methods, NWS data quality, and sample representativeness have been previously described (Monnat, 2021). Appendix Table 1 shows the distribution of NWS respondents by state. After deleting respondents who were missing on variables of interest, the analytic sample size was 3,804. Excluded respondents (N = 210) were significantly more likely than those who were included to meet the clinical thresholds for anxiety and depression, suggesting that our findings likely underrepresent individuals with the worst mental health.

### 2.2 Variables

We assessed four outcomes. *Worsened mental health* came from the question: “Overall, please rate how the COVID-19 pandemic has affected the following aspects of your life: Mental Health.” We dichotomized the 5-point Likert scale and coded ‘1’ for those who selected “somewhat worsened” or “substantially worsened”, and 0 for any other answer. *Sought mental health treatment* is a dichotomous item from the question: “Which of the following experiences did you have *as a result of* COVID-19 related closures and/or other social distancing protocols? – I sought treatment for anxiety of depression.” A strength of this outcome is that the causal attribution of physical distancing policies on mental health is supplied by the respondent. We also examined whether the respondent experienced *anxiety* or *depression* in the past 2 weeks using Patient Health Questionnaire (PHQ) items. *Depression* was based on the two items: “During the past two weeks, how often have you been bothered by: (1) having little interest or pleasure in doing things; and (2) feeling down, depressed, or hopeless.” *Anxiety* was based on the two items: “During the past two weeks, how often have you been bothered by: (1) feeling nervous, anxious, or on edge; and (2) not being able to control worrying.” Each item was measured on a 4-point scale from ‘0’=“not at all” to ‘3’=“nearly every day”. Each summed scale ranges from 0 to 6. Scores of 3 or higher on each scale meet the clinical threshold for anxiety and depression, so we dichotomized these measures using that cut point.

The independent variables are months of exposure to each of 12 state policies that restricted in-person interaction. Policy data are from the COVID-19 U.S. State Policy (CUSP) Database (Raifman et al., 2020). We calculated exposure as the number of days between when the policy was enacted and the date the policy ended (or date of survey completion if the policy was still in place then). To make results more interpretable, we used month as the unit of exposure in our models. Appendix Table 2 lists and describes each policy and provides information about the number of states where it was ever in effect, the percentage of respondents exposed, and the average, minimum, and maximum days of respondent exposure.

### 2.3 Statistical analysis

In the first part of the analysis, we used multivariable binary logistic regression models to estimate associations between exposure to the 12 separate policies and four mental health outcomes. This approach is similar to what has been done in other studies, in which each policy is examined separately. It provides important baseline information. We also examined an additive index of exposure to all 12 policies, where each policy was assumed to have equal weight. Standard errors were clustered at the state level because the state is the unit of the exposure.

In the second part of our analysis, we assessed how the physical distancing policies were collectively associated with mental health. Because the policies are highly correlated (as shown in Appendix Figure 1), we used a Bayesian group index modeling approach. These models account for the reality that states adopted multiple policies that restricted various types of activities in ways that might cumulatively and synergistically influence mental health. Among their strengths are superior model performance relative to other methods of calculating indices (such as principal component analysis [PCA]), estimation of relative policy weights directly from the data rather than assuming that policies have equal weights on the outcomes (as in the additive index), and creating indices that take into account correlations among the policies and with the outcome (unlike PCA, which would only account for the correlations among policies) (Wheeler et al., 2021). Details on the Bayesian group index models are in the Appendix.

We fitted two-group index models for each outcome to allow for the potential that some policies may have negative associations with the outcomes and others may have positive associations. If this were the case, the two-group index model may identify unique or stronger associations between the policies and mental health than the single-index model (i.e., a model that included all policies in the same index). Following standard practice, we grouped policies based on their bivariate association (positive or negative) with each mental health outcome (Wheeler et al., 2021).

All models controlled for respondent sex, age, race/ethnicity, educational attainment, number of people in the household, whether the respondent moved to their county of residence in the past year, rural–urban continuum (all from the NWS), and the county’s cumulative COVID-19 death rate at the time the survey was administered (U.S.A. Facts, 2020). We did not include covariates that might themselves represent pathways through which state policies might have affected mental health, such as employment, family, and educational disruptions, social interactions, health behaviors, and COVID-19 infection, as doing so would create over-adjustment bias (Rohrer, 2018).

The study was deemed exempt by the Syracuse University Institutional Review Board. The study was reported in accordance with STROBE guidelines (Appendix Table 3). Analyses were conducted in Stata 17.0 and R 4.1.3.

## 3 Results

Respondent characteristics from the NWS data are presented in Table 1. Nearly 38% of respondents reported that the COVID-19 pandemic somewhat or substantially worsened their mental health. A smaller share (18·4%) reported seeking treatment for anxiety or depression due to COVID-19 related closures and/or other physical distancing protocols. About one-third met the clinical thresholds for anxiety (32·6%) or depression (31·2%).

**Table 1:** National Wellbeing Survey Respondent Characteristics, U.S. Adults ages 25–64, February 1 – March 18, 2021.

	**N (%) or Mean (SD) Unweighted**	**% or Mean (SD) Weighted**
Worsened mental health	1456 (38.3)	37.9
Sought treatment for anxiety or depression	724 (19.0)	18.4
Meets clinical threshold for anxiety in past 2 weeks	1224 (32.2)	32.6
Meets clinical threshold for depression in past 2 weeks	1186 (31.2)	31.2
Age, y	41.0 (13.7)	41.0 (13.7)
Sex		
Male	1864 (49.0)	49.7
Female	1940 (51.0)	50.3
Race/ethnicity		
White (Non-Hispanic)	2322 (61.0)	61.2
Non-White	1482 (39.0)	38.8
Education		
Less than high school	172 (4.5)	10.0
High school graduate	966 (25.4)	27.7
Some college	1251 (32.9)	30.9
Bachelor's degree or higher	1415 (37.2)	31.5
Number of people in household	3.0 (1.8)	3.0 (1.8)
Respondent moved to current county within past year	256 (6.7)	6.8
Rural-Urban Continuum (based on county of residence)		
Large Metro	1750 (46.0)	56.5
Medium Metro	686 (18.0)	21.1
Small Metro	292 (7.7)	8.7
Large Nonmetro	362 (9.5)	5.6
Medium Nonmetro	400 (10.5)	6.7
Small Nonmetro	314 (8.3)	1.4
County-level COVID-19 mortality rate at time of survey	145.7 (78.1)	143.8 (70.9)

Notes: N = 3,804.

**Table 2:** Adjusted Odds Ratios and 95% Confidence Intervals (CIs) from Logistic Regression Models Predicting Adverse Mental Health Outcomes as a Function of One Month of Exposure to Separate COVID-19 Physical Distancing Policies, U.S. Adults ages 25–64, February 1 – March 18, 2021.

	**COVID-19 worsened mental health**	**Sought treatment for anxiety or depression due to COVID-19**	**Meets clinical threshold for anxiety in past 2 weeks**	**Meets clinical threshold for depression in past 2 weeks**
Stay at home order	1.00 (0.98, 1.01)	1.01 (0.99, 1.03)	1.02 (1.01, 1.04)	1.01 (0.99, 1.02)
Day care	1.05 (0.98, 1.12)	0.99 (0.92, 1.06)	1.04 (0.97, 1.11)	1.01 (0.95, 1.07)
Non-essential businesses	1.03 (0.93, 1.13)	1.08 (0.88, 1.32)	0.93 (0.82, 1.05)	0.95 (0.83, 1.09)
Business curfew	1.05 (1.01, 1.08)	1.06 (0.99, 1.13)	1.02 (0.97, 1.07)	1.02 (0.98, 1.06)
Restaurants	1.01 (0.98, 1.04)	1.02 (0.99, 1.05)	1.02 (1.00, 1.04)	1.00 (0.98, 1.02)
Gyms	1.01 (0.99, 1.03)	1.03 (1.00, 1.06)	1.02 (1.00, 1.03)	1.00 (0.98, 1.02)
Movie theaters	1.01 (0.99, 1.02)	1.03 (1.01, 1.05)	1.01 (0.98, 1.03)	1.00 (0.98, 1.01)
Bars	1.00 (0.99, 1.02)	1.03 (1.01, 1.06)	1.01 (0.99, 1.03)	1.00 (0.98, 1.01)
State-operated casinos	1.00 (0.98, 1.03)	1.02 (0.98, 1.07)	0.99 (0.96, 1.02)	0.99 (0.96, 1.01)
Hair salons	1.02 (0.97, 1.06)	1.06 (0.99, 1.13)	1.03 (0.98, 1.08)	0.99 (0.95, 1.04)
Non-essential retail	1.11 (1.02, 1.20)	1.11 (0.96, 1.28)	0.99 (0.87, 1.12)	0.96 (0.85, 1.09)
Religious gatherings	1.00 (0.95, 1.06)	1.07 (0.99, 1.16)	1.02 (0.94, 1.10)	1.01 (0.95, 1.07)
Combined policy days	1.00 (1.00, 1.01)	1.01 (1.00, 1.01)	1.00 (1.00, 1.01)	1.00 (1.00, 1.00)

Note: N = 3,804. CI = 95% confidence intervals. All models adjust for respondent sex, age, race/ethnicity, educational attainment, number of people in the household, whether the respondent moved to their current county of residence in the past year, rural–urban continuum (all from the NWS), and the county’s cumulative COVID-19 death rate at the time the survey was administered. Standard errors are clustered at state level.

**Table 3:** Adjusted Odds Ratios and 95% Credible Intervals (CRI) from Bayesian Group Index Models Predicting Adverse Mental Health Outcomes as a Function of Overall Policy Index, U.S. Adults ages 25–64, February 1 – March 18, 2021.

	**OR**	**95% CRI**	**OR**	**95% CRI**
	Index 1		Index 2	
COVID-19 Worsened Mental Health	1.36	(1.01, 1.89)	0.75	(0.50, 1.02)
Sought Treatment for Anxiety or Depression	1.15	(1.02. 1.49)	0.86	(0.59, 1.05)
Anxiety	1.06	(0.99, 1.16)	0.94	(0.77, 1.05)
Depression	1.02	(0.97, 1.07)	0.97	(0.86, 1.05)

*Notes*: N = 3,804. CRI = 95% credible intervals. Index 1 includes policies that were positively correlated with the outcome (univariate). Index 2 includes policies that were negatively correlated with the outcome (univariate).

### 3.1 Examination of separate policies

Results from the first part of our analysis, where we examined associations between exposure to each policy and mental health, are presented in Table 2. We found several significant associations. A one month increase in business curfews was associated with 5% greater odds of reporting that COVID-19 worsened one’s mental health (adjusted odds ratio [AOR] = 1·05; confidence interval [CI] = 1·01 to 1·08; p<·01). A one-month increase in non-essential retail closures was associated with 11% greater odds of this outcome (AOR = 1·11; CI = 1·02 to 1·20; p<·05). A one month increase in gym closures (AOR = 1·03; CI = 1·00 to 1·06; p<·05), movie theaters (AOR = 1·03; CI = 1·01 to 1·05; p<·01), and bars (AOR = 1·03; CI = 1·01 to 1·06; p<·01) were each associated with 3% greater odds of seeking treatment for anxiety or depression. The simple additive policy index was also associated with increased odds of seeking treatment (AOR = 1·01; CI = 1·00 to 1·01; p<·05). A one month increase in stay-at-home orders (AOR = 1·02; CI = 1·00 to 1·04, p<·05) and restaurant closures (AOR = 1·02; CI = 1·01 to 1·04, p<·01) were both associated with 2% greater odds of meeting the clinical threshold for anxiety.

Although the magnitude of these ORs may seem small, these policies were in effect for longer than one month for most respondents. For example, NWS respondents experienced an average of 114 days (3·75 months) of gym closures, 144 days (4·73 months) of movie theater closures, and 184 days (6·05 months) of bar closures, and some respondents were exposed to these policies for the entire year preceding the survey (see Appendix Table 2).

### 3.2 Examination of policy bundles

**Fig. 1:**
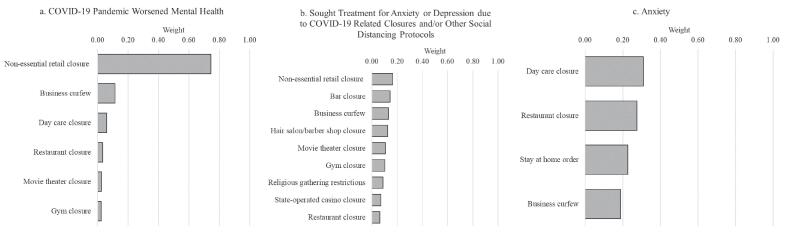
**Weights for Policies Positively Correlated with a) Worsened Mental Health, b) Sought Treatment for Anxiety or Depression, and c) Anxiety, U.S. Adults ages 25**–**64, February 1 – March 18, 2021***Note*: Weights sum to 1. We do not show a figure for depression because business curfew is the only policy to load onto that index.

Results from the Bayesian two-group index models are presented in Table 3. Index 1 for each mental health outcome includes the policies that were positively correlated with that outcome. Index 2 includes the policies that were negatively correlated with the outcome. None of the associations for Index 2 were statistically significant. Therefore, we focus on the results for Index 1. We show the weights for each policy included in Index 1 for each outcome in Fig. 1 (weights for each index sum to 1).

The six policies included in the index that was associated with greater odds of worsened mental health were non-essential retail closure, business curfew, day care closure, restaurant closure, movie theater closure, and gym closure. The nine policies in the index associated with greater odds of seeking treatment were non-essential retail closure, bar closure, business curfew, hair salon/barber shop closure, movie theater closure, gym closure, religious gathering restrictions, state-operated casino closure, and restaurant closure. The four policies in the index associated with greater odds of anxiety were day care closure, restaurant closure, stay-at-home order, and business curfew. Only one policy – business curfew – loaded into Index 1 for depression.

The adjusted odds ratios (Table 3) show that a one-month increase in each outcome’s respective index was associated with a 36% increase in the odds of reporting worsened mental health (AOR = 1·36; 95% credible interval [CRI] = 1·01 to 1·80), a 15% increase in the odds of seeking treatment for anxiety or depression (AOR = 1·15; CRI = 1·02 to 1·49), and a 6% increase in the odds of meeting the clinical threshold for anxiety (OR = 1·06; CRI = 0·99 to 1·16). The index for depression (comprised only of business curfew) was associated with a 2% increase in the odds of depression (OR = 1·02, CRI = 0·97 to 1·07).

In terms of the relative contribution of each policy to the indices (see Fig. 1), for worsened mental health (panel a), non-essential retail closures contributed the most weight by far (0·744 in an index where weights sum to 1). All nine policies in the index predicting seeking treatment for anxiety or depression (panel b) had similar weights (ranging from 0·063 to 0·164), indicating that they all played similarly important roles. For anxiety (panel c), all four policies in the index—day care closures, restaurant closures, stay at home orders, and business curfews—had sizable weights (ranging from 0·188 to 0·310), indicating that they all played important roles.

## 4 Discussion

This study considered how a dozen state COVID-19 physical distancing policies were associated, both individually and collectively, with mental health outcomes among working-age (18–64) adults one year into the pandemic. There are several important findings.

First, we found that several individual physical distancing policies were associated with greater odds of reporting that COVID-19 worsened one’s mental health, seeking treatment for anxiety or depression as “a result of COVID-19 related closures and/or other social distancing protocols”, and meeting the clinical threshold for anxiety. Business curfews, non-essential retail closures, gym closures, movie theatre closures, restaurant closures, bar closures, and stay-at-home orders were all associated with significantly greater odds of at least one of these mental health outcomes. The magnitude of these associations for a one month increase in exposure to a specific policy ranged from 1% to 11% greater odds of reporting the respective adverse mental health outcome. While these findings provide important baseline information, they tell us only how single policies were associated with mental health. In reality, states enacted multiple policies. Therefore, our approach extended these baseline analyses by using Bayesian group index models to consider how exposure to multiple policies (“policy bundles”) predicted mental health.

Our second main takeaway is that several policies bundled together to predict even larger associations with these mental health outcomes, and these models provide us with superior information beyond our analyses of individual policies. Specifically, these models revealed that nearly all policies (11 of the 12) were significantly associated with mental health outcomes. This contrasts with the models that examined the policies separately, which did not detect any association between mental health outcomes and day care closures, hair salon/barber shop closures, restrictions on religious gatherings, and closures of state-operated casinos. These novel findings imply that COVID-era physical distancing policies may have had synergistic relationships with mental health in ways that cannot be observed when considering policies individually. They also imply that using stay-at-home orders as a proxy for business closures (Tull et al., 2020, Marroquín et al., 2020, Barrett et al., 2021) may underestimate the relationship between states’ physical distancing policies and adult mental health. Moreover, our index models revealed much larger associations between exposure to the policy bundles and the mental health outcomes than what we observed for associations between individual policies and outcomes. Overall, we show a 36% increase in odds of reporting worsened mental health, a 15% increase in odds of seeking treatment for anxiety or depression, and a 6% increase in odds of meeting the clinical threshold for anxiety for a one-month increase in exposure to the indices of physical distancing policies. Ultimately, our findings suggest that accurately assessing the relationship between states’ COVID-era policies and working-age adult mental health requires understanding that people experience more than one policy at a time (Beckfield et al., 2015).

Third, our index modeling approach extends prior research by enabling us to identify the policies that had the strongest associations with mental health outcomes (by identifying the weight each policy contributed to the indices). While eleven policies contributed in at least some statistically significant way to the mental health outcomes, business curfews, non-essential retail closures, and restaurant closures appeared to make the largest contributions both in terms of their presence in multiple indices (e.g., business curfews and restaurant closures appeared in all three of the significant indices) and in terms of the weights attributed to those policies in the indices. Whereas non-essential retail closures dominated the index associated with worsened mental health, the policies that contributed to the indices for seeking treatment for mental health and for meeting the clinical threshold for anxiety made relatively equal contributions within the respective indices. This suggests that there was not one single policy that drove the adverse mental health outcomes observed in this study. Instead, policies cumulatively and synergistically contributed to adverse mental health one year into the pandemic. It is important to recall that most states had rescinded their physical distancing policies many months prior to NWS data collection. Our findings suggest potentially long-lasting adverse mental health consequences of physical distancing policies, beyond the long-term effects of the pandemic at which they were targeted.

As noted in the introduction, there are several potential explanations for our observed relationships between physical distancing policies and adverse mental health outcomes. It was beyond the scope of this study to identify the role of mediators. Now that we have established associations between bundles of COVID-19 policies and adverse mental health outcomes, we encourage future research to identify the mechanisms linking exposure to multiple policies to these outcomes.

The strengths of this study include the use of a large, geocoded sample of working-age adults who answered questions about their mental health while the U.S. was still in the midst of the COVID-19 pandemic, the simultaneous consideration of a dozen state physical distancing policies on four mental health outcomes, and the use of a modelling approach that enabled us to consider how sets (“bundles”) of correlated policies that restricted different types of activities were associated with mental health and which policies contributed the most weight to different outcomes.

Despite its strengths, the study is subject to limitations. First, the data are cross-sectional and capture one point in time during the pandemic. Although one outcome is based on a question where respondents placed the causal attribution on social distancing policies, causality should not be presumed given that we do not have baseline measures of mental health and cannot assess how it changed from before policies were implemented. Second, we cannot definitively conclude whether the associations we found between policies and mental health were due to the policies themselves, above and beyond the general effects of the pandemic. Although we controlled for cumulative COVID-19 mortality rates, there could be other factors that were correlated with the policies that also affected mental health. Third, although the sample is demographically representative, it may not be representative of COVID-19 impacts. For example, those who were the most negatively affected by COVID-19 may have been less likely to complete the survey. Previous research using the NWS found strong comparability between several univariate estimates from the NWS and other national household surveys collected during the same time frame (Monnat, 2021), and the NWS prevalence rates we found for anxiety and depression are in line with rates from the U.S. Census Household Pulse from April 2020 to March 2021 (Coley et al., 2022). In addition, we did not include state school closure policies because nearly all states closed schools in the first weeks of the pandemic, which was typically followed by a long summer break. Reopening policies in Fall 2020 varied district-by-district within states. Given the extreme stressors associated with caring for and homeschooling children (Czeisler et al., 2021); (Frank et al., 2021) and its similarity with day care closures, we would expect to find that such a policy increased anxiety.

## 5 Conclusion

Our results have public health implications beyond the policies and mental health outcomes we considered here. One implication is that there are tradeoffs between mitigation policies designed to combat disease spread and individual mental health. Moreover, it is important to recognize the potentially distinct roles of short- and long-term consequences of interventions. However successful were the interventions aimed at controlling COVID-19 spread—which might be quantified by case counts, hospitalizations, and deaths—the harder-to-quantify and possibly delayed mental health consequences should be considered as well. Assessing the costs, benefits, and temporal dynamics of these policies more fully is an important avenue for future research. We also showed the importance for policymakers to understand the role of multiple policy changes on mental health outcomes. People live in “more than one policy at a time” (Beckfield et al., 2015). Public health decision makers must consider both the benefits and harms of any policy and implement those that maximize the former and minimize the latter.

## 6 Funding declaration

This project was funded by the National Institute on Drug Abuse (U01DA055972). The funders played no role in the writing of the manuscript or decision to submit it for publication. The authors have no conflicts of interest to declare.

## Declaration of Competing Interest

The authors declare that they have no known competing financial interests or personal relationships that could have appeared to influence the work reported in this paper.

## Data Availability

State policy data are publicly available from the COVID-19 U.S. State Policy Database (https://statepolicies.com/). National Wellbeing Survey data are available through the Inter-university Consortium for Political and Social Research (ICPSR) via a data use agreement.
