# Supplementary material for: U.S. States’ COVID-19 physical distancing policies and working-age adult mental health outcomes
Source: Prev Med Rep. 2023 Aug 18;35:102370. doi: 10.1016/j.pmedr.2023.102370 (PMC10468353; doi:10.1016/j.pmedr.2023.102370)
Supplement: Supplementary data 1 [file mmc1.docx]

**U.S. States’ COVID-19 Physical Distancing Policies and**

**Working-Age Adult Mental Health Outcomes**

**APPENDIX**

**Appendix Table 1. Distribution of National Wellbeing Survey Respondents by State, U.S. Adults ages 25-64, February 1 – March 18, 2021**

| **State** | **N Respondents** | **Unweighted %** | **Weighted %** |
| --- | --- | --- | --- |
| Alabama | 64 | 1.68 | 1.45 |
| Alaska | 10 | 0.26 | 0.13 |
| Arizona | 71 | 1.87 | 2.30 |
| Arkansas | 61 | 1.60 | 1.12 |
| California | 346 | 9.10 | 10.49 |
| Colorado | 53 | 1.39 | 1.40 |
| Connecticut | 53 | 1.39 | 1.62 |
| Delaware | 16 | 0.42 | 0.59 |
| District of Columbia | 10 | 0.26 | 0.30 |
| Florida | 243 | 6.39 | 7.34 |
| Georgia | 164 | 4.31 | 4.07 |
| Hawaii | 26 | 0.68 | 0.58 |
| Idaho | 29 | 0.76 | 0.51 |
| Illinois | 141 | 3.71 | 3.82 |
| Indiana | 73 | 1.92 | 2.10 |
| Iowa | 41 | 1.08 | 0.79 |
| Kansas | 38 | 1.00 | 0.78 |
| Kentucky | 95 | 2.50 | 1.32 |
| Louisiana | 51 | 1.34 | 1.52 |
| Maine | 16 | 0.42 | 0.39 |
| Maryland | 64 | 1.68 | 1.93 |
| Massachusetts | 46 | 1.21 | 1.59 |
| Michigan | 115 | 3.02 | 2.73 |
| Minnesota | 34 | 0.89 | 0.96 |
| Mississippi | 48 | 1.26 | 0.81 |
| Missouri | 79 | 2.08 | 1.83 |
| Montana | 16 | 0.42 | 0.28 |
| Nebraska | 30 | 0.79 | 0.41 |
| Nevada | 33 | 0.87 | 1.11 |
| New Hampshire | 13 | 0.34 | 0.32 |
| New Jersey | 107 | 2.81 | 3.33 |
| New Mexico | 22 | 0.58 | 0.48 |
| New York | 293 | 7.70 | 8.48 |
| North Carolina | 131 | 3.44 | 3.10 |
| North Dakota | 11 | 0.29 | 0.18 |
| Ohio | 137 | 3.60 | 3.91 |
| Oklahoma | 55 | 1.45 | 1.53 |
| Oregon | 41 | 1.08 | 1.06 |
| Pennsylvania | 137 | 3.60 | 3.78 |
| Rhode Island | 11 | 0.29 | 0.36 |
| South Carolina | 61 | 1.60 | 1.69 |
| South Dakota | 22 | 0.58 | 0.27 |
| Tennessee | 100 | 2.63 | 2.59 |
| Texas | 288 | 7.57 | 7.67 |
| Utah | 19 | 0.50 | 0.60 |
| Vermont | 5 | 0.13 | 0.05 |
| Virginia | 110 | 2.89 | 2.51 |
| Washington | 62 | 1.63 | 1.56 |
| West Virginia | 37 | 0.97 | 0.61 |
| Wisconsin | 61 | 1.60 | 1.41 |
| Wyoming | 15 | 0.39 | 0.23 |

*Notes*: N=3,084

**Appendix Table 2. Definitions and Descriptive Statistics for Respondent Exposure to State Physical Distancing Policies Included in the Study, U.S. Adults ages 25-64, February 1 – March 18, 2021**

|  |  |  |  | **Respondent Exposure to Policy (Days)** | | |
| --- | --- | --- | --- | --- | --- | --- |
| **Policy** | **Definition** | **N States** | **% Respondents got exposed** | **Avg.** | **Min.** | **Max** |
| Day care | Number of days the state mandated daycare closures^a^ | 15 | 27.1 | 19.8 | 36 | 90 |
| Non-essential businesses | Number of days the state mandated closures of non-essential businesses^a^ | 50 | 99.42 | 48.2 | 20 | 79 |
| Business curfew | Number of days the state mandated closure of businesses for certain hours overnight | 25 | 48.84 | 38.1 | 1 | 218 |
| Restaurants | Number of days the state mandated closure of restaurants (except for takeout)^a^ | 50 | 99.42 | 92.4 | 25 | 312 |
| Gyms | Number of days the state mandated closure of indoor gyms/fitness centers^a^ | 50 | 99.42 | 114.1 | 22 | 364 |
| Movie theaters | Number of days the state mandated closure of movie theaters^a^ | 50 | 99.42 | 143.8 | 25 | 364 |
| Bars | Number of days the state mandated closure of bars statewide^a^ Unless otherwise noted, bars are defined as establishments that derive more than 50 percent of gross revenue from the sales of alcoholic beverages. | 50 | 99.42 | 183.9 | 43 | 367 |
| State-operated casinos | Number of days the state mandated closure of state-operated casinos^a^ Order must apply to the entire state excepting sovereign territories. | 23 | 42.25 | 50.6 | 19 | 360 |
| Hair salons | Number of days the state mandated closure of hair salons and barber shops^a^ | 50 | 99.42 | 66.9 | 22 | 163 |
| Religious gathering restrictions | Number of days state mandated closure of religious gatherings with exemption for social distancing mandates. A clear social distancing requirement is defined as a mandate that gatherings must be less than 10 people and/or adherence to CDC social distance guidelines. Must be statewide. | 37 | 60.38 | 36.5 | 30 | 93 |
| Non-essential retail | Number of days the state mandated closure of non-essential retail statewide. | 50 | 99.42 | 51.5 | 20 | 101 |
| Stay at home order | Number of days a state stay-at- home/shelter in place order was in effect^a^ | 44 | 94.77 | 76.7 | 25 | 313 |

*Notes*: Data in the table are based on policies that were in effect starting in March 2020 and up until and between February 1 and March 18, 2021 (the period of the NWS collection); CUSP codebook: <https://statepolicies.com/data/library/codebook/>*.* The average includes 0s for respondents living in states without the policy. The minimum excludes 0s and instead reports the minimum days of exposure only for respondents in states that ever had the policy in effect. Nearly all of the policies we consider were first implemented during a short window (approximately three weeks), such that the duration of exposure is also representative of the recency of policy discontinuation (i.e., policies with longer duration were discontinued more recently than those with shorter duration).

^a^ Only included directives/orders. Did not include guidance or recommendations. Order must apply to entire state.

**Appendix Table 3. STROBE checklist**

|  | Item No | Recommendation | Location where item is reported |
| --- | --- | --- | --- |
| **Title and abstract** | 1 | (*a*) Indicate the study’s design with a commonly used term in the title or the abstract | Abstract: Methods |
|  |  | (*b*) Provide in the abstract an informative and balanced summary of what was done and what was found | Abstract: Methods  Abstract: Findings  Abstract: Interpretation |
| Introduction | | |  |
| Background/rationale | 2 | Explain the scientific background and rationale for the investigation being reported | Research in context  Introduction |
| Objectives | 3 | State specific objectives, including any prespecified hypotheses | Research in context  Introduction |
| Methods | | |  |
| Study design | 4 | Present key elements of study design early in the paper | Methods  Appendix |
| Setting | 5 | Describe the setting, locations, and relevant dates, including periods of recruitment, exposure, follow-up, and data collection | Methods |
| Participants | 6 | (*a*) Give the eligibility criteria, and the sources and methods of selection of participants | Methods |
| Variables | 7 | Clearly define all outcomes, exposures, predictors, potential confounders, and effect modifiers. Give diagnostic criteria, if applicable | Methods |
| Data sources/ measurement | 8* | For each variable of interest, give sources of data and details of methods of assessment (measurement). Describe comparability of assessment methods if there is more than one group | Methods |
| Bias | 9 | Describe any efforts to address potential sources of bias | Methods |
| Study size | 10 | Explain how the study size was arrived at | Methods |
| Quantitative variables | 11 | Explain how quantitative variables were handled in the analyses. If applicable, describe which groupings were chosen and why | Methods |
| Statistical methods | 12 | (*a*) Describe all statistical methods, including those used to control for confounding | Methods  Appendix |
|  |  | (*b*) Describe any methods used to examine subgroups and interactions | Methods |
|  |  | (*c*) Explain how missing data were addressed | Methods |
|  |  | (*d*) If applicable, describe analytical methods taking account of sampling strategy | Methods |
|  |  | (*e*) Describe any sensitivity analyses | Methods |
| Results | | |  |
| Participants | 13* | (a) Report numbers of individuals at each stage of study—eg numbers potentially eligible, examined for eligibility, confirmed eligible, included in the study, completing follow-up, and analysed | Results |
|  |  | (b) Give reasons for non-participation at each stage | NA |
|  |  | (c) Consider use of a flow diagram | NA |
| Descriptive data | 14* | (a) Give characteristics of study participants (eg demographic, clinical, social) and information on exposures and potential confounders | Results  Appendix |
|  |  | (b) Indicate number of participants with missing data for each variable of interest | All missing was excluded. |
| Outcome data | 15* | Report numbers of outcome events or summary measures | Results |
| Main results | 16 | (*a*) Give unadjusted estimates and, if applicable, confounder-adjusted estimates and their precision (eg, 95% confidence interval). Make clear which confounders were adjusted for and why they were included | Results |
|  |  | (*b*) Report category boundaries when continuous variables were categorized | Results |
|  |  | (*c*) If relevant, consider translating estimates of relative risk into absolute risk for a meaningful time period | NA |
| Other analyses | 17 | Report other analyses done—eg analyses of subgroups and interactions, and sensitivity analyses | Results |
| Discussion | | |  |
| Key results | 18 | Summarise key results with reference to study objectives | Discussion |
| Limitations | 19 | Discuss limitations of the study, taking into account sources of potential bias or imprecision. Discuss both direction and magnitude of any potential bias | Discussion |
| Interpretation | 20 | Give a cautious overall interpretation of results considering objectives, limitations, multiplicity of analyses, results from similar studies, and other relevant evidence | Discussion |
| Generalisability | 21 | Discuss the generalisability (external validity) of the study results | Discussion |
| Other information | | |  |
| Funding | 22 | Give the source of funding and the role of the funders for the present study and, if applicable, for the original study on which the present article is based | Methods |

*Give information separately for exposed and unexposed groups.

*Note:* An Explanation and Elaboration article discusses each checklist item and gives methodological background and published examples of transparent reporting. The STROBE checklist is best used in conjunction with this article (freely available on the Web sites of PLoS Medicine at http://www.plosmedicine.org/, Annals of Internal Medicine at http://www.annals.org/, and Epidemiology at http://www.epidem.com/). Information on the STROBE Initiative is available at www.strobe-statement.org.

**Appendix Figure 1. Correlations between State Physical Distancing Policy Exposure Months among NWS Respondents, U.S. Adults ages 25-64**

*Note*: The plot represents correlations between the 12 state policies measure by respondent exposure in months.

Details about Bayesian group index Regression Model

The Bayesian group index regression model for a binary health outcome $y_{i}\sim\mathrm{Bernoulli}(p_{i})$ and groups of policies is specified through the log-odds for the *i*th respondent as:

| , |  |
| --- | --- |

where the left-hand side of the equation is the logit of the outcome probability $p_{i}$, $w_{jk}$ is the weight parameter for the$j\text{th}$policy in the $k\text{th}$group with exposure months $q_{ijk}$ for the *i*th individual, $\beta_{k}$ is the coefficient for the $k\text{th}$index, and $z_{i}^{T}$ is a vector of covariates with corresponding coefficients in vector . Each weighted index has $C_{k}$ number of policies. The weights represent the relative importance of the policy exposures and are constrained to be between 0 and 1 and to sum to 1 for each index. This model can identify the most important group of policies through posterior inference on the index coefficients $\beta_{k}$ and the most important variables in each group through posterior inference on the weights. The model can be simplified to contain only one index, which is referred to as a Bayesian index model or Bayesian single index model.

Assignment of prior distributions for the model parameters completes the model specification. The index weights $w_{1k},\ldots,w_{C_{k}}$ are given a Dirichlet prior with parameters $\mathbf{\alpha}=(\alpha_{1},\ldots,\alpha_{C})$. The Dirichlet prior is convenient because it assures that the weights $w_{j}\in(0,1)$ and $\sum_{j=1}^{C_{k}} w_{jk}=1$. The intercept, index regression coefficients, and covariate regression coefficients are assigned vague normal priors, with precision $\tau_{k}=1/\sigma_{k}^{2}$ and $\sigma_{k}\sim Uniform(0,100)$. Markov Chain Monte Carlo (MCMC) is used to estimate the model parameters. Convergence of the MCMC algorithm is evaluated using the Gelman-Rubin statistic. Goodness-of-fit of the one-group and two-group models is assessed using the deviance information criterions (DIC).
